# The regulatory effects of bioactive polysaccharides on intestinal function and bile acids: chemical structures, bioactivities, and mechanisms

**DOI:** 10.3389/fnut.2024.1495993

**Published:** 2024-11-15

**Authors:** Anqi Wang, Wugui Xiong, Jing Li, Yingfan Hu, Liang Zou, Ying Liu

**Affiliations:** ^1^School of Food and Bioengineering, Institute for Advanced Study, Chengdu University, Chengdu, China; ^2^School of Preclinical Medicine, Chengdu University, Chengdu, China; ^3^Antibiotics Research and Re-Evaluation Key Laboratory of Sichuan Province, Sichuan Industrial Institute of Antibiotics, Chengdu University, Chengdu, China

**Keywords:** polysaccharides, bile acids, metabolic diseases, inflammation, gut microbiota, intestinal function

## Abstract

Polysaccharides are one of the important components of the human diet, offering a wide range of biological activities, especially in improving inflammation in the digestive system and addressing metabolic diseases. Among all the reported bioactivities of polysaccharides, their regulation effects on intestinal function and bile acids (BAs) are gradually attracting the attention of more researchers. Bile acids, the main components of intestinal lipid digestive fluid, are also key signal factors for metabolic homeostasis and impact overall health. Polysaccharides usually interact directly or indirectly with the gut and gut microbiota to participate in the regulation process of reabsorption, metabolism, and excretion of bile acids in humans, thereby exerting their role in the intervention of human diseases. In this review, we comprehensively reviewed the effects of bioactive polysaccharides on the regulation of intestinal function and bile acids. The chemical structures, bioactivities, and potential mechanisms for their activities were also reviewed. This study aimed to provide a comprehensive reference for future research on the activities and mechanisms of polysaccharides, as well as to offer important strategies and insights for the development of bioactive polysaccharides to prevent inflammatory and metabolic diseases.

## Introduction

Polysaccharides, a type of polymer formed by aldoses or ketoses with glycosidic bonds, are often composed of more than 10 monosaccharides arranged in chains and branch shapes, and monosaccharide compositions and structures are complex and diverse. Polysaccharides are widely distributed in nature and have great development and application potential. They are one of the important components of fruits, grains, and vegetables. The biological activities of polysaccharides include lowering blood sugar (1), anti-obesity (2), liver protection (3), regulating immunity (4), anti-oxidation (5), and anti-tumor effects (6). Hence, research interest in these kinds of polymers has been rapidly increasing in recent years.

The intestine plays an important role in digestion and absorption and is also a barrier that prevents harmful substances from entering the bloodstream. Intestinal lymphocytes often secrete inflammatory mediators and cytokines to regulate the intestinal immune environment (7). In addition, the intestinal barrier function often affects intestinal-liver metabolism. When the intestinal barrier function is changed, it can easily induce obesity (8), type 2 diabetes (9), asthma (10), rheumatoid arthritis (11), and other diseases in humans. Metabolites in the intestine (such as intestinal D-lactate) can enter the bloodstream through damaged intestinal mucosa and participate in regulating various metabolic reactions in the body, serving as an important biological barrier for intestinal anti-infection. Bile acids (BAs) play an important role in maintaining the homeostasis of the bile pool (12), improving metabolic inflammation (13), and providing neuroprotection (14). Meanwhile, the Farnesoid X receptor (FXR) is a bile acid-activated nuclear receptor mainly expressed in the liver and intestine. Upon stimulation by specific bile acids for the FXR, key genes involved in bile acid synthesis, transport, and reabsorption metabolism are regulated and participate in the regulation of carbohydrate and lipid metabolism (15). Hence, it seems that targeting the regulation of intestinal function and bile acid metabolism might be valuable for the treatment of various diseases. Therefore, focusing on the regulatory effects of bioactive polysaccharides on intestinal function and bile acid metabolism is important for future research.

For most orally administrated polysaccharides, they are firstly and widely exposed to the gastrointestinal tract. Therefore, their biological activities in regulating intestinal function may involve the following aspects: (1) polysaccharides may promote the proliferation of beneficial bacteria and/or inhibit the growth of pathogenic bacteria, thereby affecting the metabolism of drugs and food by gut microbiota and presenting various biological activities (16). (2) Polysaccharides may enhance intestinal barrier function and present activities in regulating immune, anti-inflammatory, and other related biological functions (17). (3) Polysaccharides may regulate the digestion and absorption of food by affecting the activity of intestinal digestive enzymes, which may be beneficial for relieving constipation and other related diseases (18).

Apart from the interactions between polysaccharides and the gut, the regulation of the bile acid pool by polysaccharides also has some effects on human health. Bile acids, an important component of bile, are often considered to promote the digestion and absorption of lipids and fat-soluble vitamins in the intestine. Furthermore, evidence also demonstrates that these components are important metabolic regulatory signal factors in the human body, widely involved in the metabolism of glucose, lipids, and energy (19). Three pathways may be involved in the connections between the biological activities of polysaccharides and bile acids: 1) polysaccharides may inhibit the absorption of fat, cholesterol, and bile acids in the intestine through physical or chemical combinations, and thus the excretion of fat, cholesterol, and bile acids is increased, which leads to an increase in the content of blood lipids and a reduction in the risk of cardiovascular diseases. 2) Polysaccharides directly combine with bile acids to inhibit their reabsorption, increase their excretion, regulate bile acids in other ways, and exert biological activities, such as hypoglycemic, hypolipidemic, and hepatoprotective. 3) Polysaccharides regulate the expression of bile acid synthase, metabolic enzymes, and transporters by activating a variety of nuclear receptors in the intestines. The changes in bile acids in the intestine contribute significantly to the changes in gut microbiota structure and, in turn, affect the metabolism of bile acids at all levels. The regulatory effects of bioactive polysaccharides on intestinal function and bile acids are briefly listed in Table 1.

**Table 1:** Summary of the regulatory effects of bioactive polysaccharides on intestinal function and bile acids.

No	Source of Polysaccharides	Dosage for animals	Chemical composition	Biological activities	Mechanisms	References
1	*Arctium lappa*	300 mg/kg, gavage for 25 days	Inulin-neoseries fructan (neokestose) is composed of (2→1)-β-D-Fruf linked to a terminal (2→1)-α-d-glucopyranose at the non-reducing end, with (2→6)-β-D-Fruf branching on its backbone	Alleviation of systemic inflammation induced by intraperitoneal injection of lipopolysaccharide in mice	Increase the abundance of *Lactobacillius, Alistipes, Odoribacter, and Phascolarctobacterium* and reduce the abundance of *Bacteroides*	(49, 74)
2	Flaxseed	50–150 g/kg, gavage for 8 weeks	/	Reduce fat accumulation and the occurrence and development of metabolic syndrome induced by a high-fat diet	Increase the proportion of *Ackermann* and *Bifidobacterium* and decrease the abundance of *Oscillospira* and *Odoribacteraceae*	(51, 52)
3	*Dendrobium officinale*	50–200 mg/kg, gavage for 6 weeks	Composed of mannose, glucose, and arabinose, with a molar ratio of 5.55:1:0.12 (average molecular weight, 393.8 kDa)	Recover intestinal barrier function and reduce inflammation injury induced in colitis rats	Enhance the expression of ZO-1 and occludin in the intestine	(55)
4	*Dimocarpus longan*	100–400 mg/kg, gavage for 4 weeks	A high molecular weight (1.47 × 10^5^ Da) and two specific glycosidic linkages of α-Araf-(1→ and →5)-α-Araf-(1→.	Protect intestinal mucosal from injury by cyclophosphamide	Increase the expression of ZO-1, claudin-1, occludin, and adhesion junction protein E-cadherin	(57)
5	*Spirulina platensis*	50–200 mg/kg, gavage for 1 week	Composed of the →2)-α-L-Rhap-(1→, →4)-β-D-Manp-(1→, →6)-β-D-Glcp-(1→, →4)-β-Xylp-(1→, →3)-β-L-Araf-(1→, and →2)-β-L-Fucp-(1→, respectively	Relieve constipation in mice induced by diphenoxylate	Restore intestinal xylanase and protease activities to normal levels in mice	(58, 59)
6	*Dendrobium officinale*	560 mg/kg, gavage for 1 week	Mainly composed of glucose and mannose (Manp:Glcp = 2.01:1.00–8.82:1.00), along with galactose, xylose, arabinose, and rhamnose in different molar ratios	Improve spleen deficiency constipation	Restore intestinal xylanase, amylase, and protease activities, and decrease intestinal cellulase activity in mice	(60, 61)
7	*Eriobotrya japonica*	*In vitro* intestinal simulation experiment	Composed of rhamnose, galacturonic acid, arabinose, and galactose, with a molecular weight of 4.307 ~ 5.101 × 10^6^ Da	Potential effects for preventing obesity and type 2 diabetes	High affinity to fat/cholesterol and bile acids *in vitro*	(52)
8	*Abelmoschus esculentus*	*In vitro* bile acids binding experiment	/	Increase the excretion of fat, cholesterol, and bile acids	High binding capacity to fat/cholesterol and bile acids	(62)
9	*Caulerpa lentillifera*	*In vitro* bile acids binding experiment	/	Inhibit the reabsorption and excretion of bile acids	High binding capacity to cholic acid, deoxycholic acid, glycocholic acid, and taurocholic acid	(63)
10	*Laminaria japonica*	*In vitro* bile acids binding experiment	Highly-branched structure, abundant sulfate, fucose, and galactose in chemical composition, and a denser interconnected macromolecule network in the molecular morphology of polysaccharides	/	Strong binding ability to cholic acid, glycocholic acid, and taurocholic acid	(64)
11	*Momordica charantia*	*In vitro* bile acids binding experiment	Composed of arabinose, galactose, glucose, xylose, and mannose	/	Strong binding capacity to cholic acid and chenodeoxycholic acid *in vitro*	(65)
12	Stigma Maydis	*In vitro* and *vivo* bile acids binding experiment	Composed of D-mannose, L-rhamnose, D-glucose, D-galactose, L-arabinose, D-xylose, and D-galacturonic acid, with a molar ratio of 1.00:0.21:1.41:1.44:0.70:0.44:0.56 bonded by (1→6) and (1→3) linkages, with various monosaccharides distributed in the main and side chains	Lower the total cholesterol, triglyceride, and low-density lipoprotein cholesterol levels in hyperlipidemic mice	Strong binding capacity to both taurocholic acid sodium and glycodeoxycholic acid sodium	(66)
13	Apple pectin	/	/	Increase the excretion of bile acids in feces and decrease serum cholesterol levels	Increase the expression of the FXR and bile acid transporters ASBT and MRP2 in the ileum and the FXR, OSTα/β, and MRP3 in the cecum	(67)
14	Haw pectin penta-oligogalacturonide	300 mg/kg BW, 4 weeks	/	Decrease cholesterol accumulation and improve metabolism of cholesterol	Reduce the expression of the FXR in the intestine of mice and suppress intestinal bile acid reabsorption	(68)
15	Cellulose from sweet potato residues	100 mg/kg BW, 28 days	/	Improve lipid metabolism in hypolipidemic rats	Increase the ileal apical sodium-dependent bile acid transporter and intestinal bile acid binding protein	(69)
16	*Grifola frondosa*	900 mg/kg, 8 weeks	/	Increase the hepatic expression of the bile acid synthase CYP7A1 and the bile salt export pump (BSEP) in diabetic mice	Positively correlate with increased abundances of *Roseburia*, *Lachnoclostridium*, *Lachnospiraceae*_*NK4AB6_group*, *Rikenella*, *Bacteroides,* and *Alistipes* and decreased abundances of *Streptococcus*, *Staphylococcus*, *Enterococcus,* and *Aerococcus*	(70)
17	*Gracilaria lemaneiformis*	60 mg/kg, 6 weeks	Composed of galactose, glucose, fucose, and mannose, with a molar ratio of 9.16: 6.57: 1.00: 0.61, and the glycosidic linkages are 84→3,4)-Fucp-(1→, →3,4,6)-Galp-(1→, →4)-Glcp-(1→, →4,6)-Manp-(1→, →6)-Glcp-(1→,85→6)-Galp-(1→ and Galp-(1→ with the ratio of 0.36: 1.36: 0.26: 0.13: 36.58: 54.36: 6.96	Accelerate the conversion of cholesterol to bile acids in high-fat fed mice	Increase abundances of *Lachnospiraceae_NK4A136_group* and *Roseburia,* and decrease abundances of *Alistipes*, *Prevotellaceae*_*UCG-001,* and *Corprococcus*	(71)
18	*Chlorella pyrenoidosa*	150–300 mg/kg, 8 weeks	Composed of 1,2-linked-α-L-Fucp, 1,4-linked-α-L-Rhap, 1,4-linked-β-L-Araf, 1-linked-α-D-Glcp, 1,3-linked-β-D-GlcpA, 1,4-linked-β-D-Xylp, and 1,3,6-linked-β-D-Manp, with a molecular weight of 5.63 × 10^6^ Da	Accelerate the metabolism of cecal total bile acids and short-chain fatty acids in hyperlipidemia rats	Increase abundances of *Ruminococcus*_1, *Coprococcus*_1, *Peptococcus,* and *Acetatifactor*.	(72)
19	Inulin-type fructans	250 mg/mouse/day, 2 weeks	/	Improve endothelial dysfunction in mice with vascular dysfunction	Increase abundances of *Akkermansia, Ruminococcaceae,* and *Lachnospiraceae*	(73)

In this review, the recent research progress on the regulatory effects of bioactive polysaccharides on intestinal function and bile acids is comprehensively summarized. Focusing on the research progress of polysaccharides in regulating bile acid synthesis, reabsorption, metabolism, and excretion through intestinal action is expected to provide a foundational basis for studying the regulatory mechanisms by which polysaccharides exert various bioactivities in the human body.

## Regulation of intestinal function by gut mucosa and microbiota

The intestinal mucosa consists of gut microbiota, the mucus layer, intestinal epithelial cells, and immune barriers in the gut, which act between the gut and the external environment and work together to prevent harmful bacteria, toxins, and other external stimuli from entering the gut (20). The highly glycosylated, polymerized mucins on the surface of epithelial cells serve as the first line of defense against physical and chemical damage in the gut. These mucins are rich in serine and threonine protein backbone structure and are linked with multiple O-linked oligosaccharide side chains (21, 22). Intestinal goblet cells not only secrete mucins to create a mechanical protective barrier for intestinal homeostasis but also contribute to the intestinal immune system barrier, which helps maintain intestinal health (23). In addition, some studies have also found that changes in the structure and function of mucins are closely related to the onset and progression of intestinal inflammation and other intestinal diseases (24).

The gut microbiome plays an important role in the development of pathogen resistance, mucosal immune responses, and nutrient metabolism. This is partly due to the interaction between the microbiota and the composition of the mucus layer and intestinal epithelial cells following damage to the mucus. The outer permeable mucus layer is considered the natural habitat of many symbionts because they use exposed mucins as attachment sites for nutritional support and bacterial adhesions (25). Some enzymes produced by gut bacteria are associated with the digestion of various glycans from the mucus and fibers from the host’s diet. The mucus regulatory effects of certain commensals, probiotics, especially *Lactobacillus* and *Bifidobacterium* strains, and probiotic mixtures have been demonstrated in animal models of IBD, reproductive disorders, diet-induced obesity, malnutrition, and aging (26–29). Taken together, these results suggest that regulating intestinal mucosal barrier function and gut microbiota may provide benefits for the prevention and treatment of a variety of diseases. Therefore, the regulatory effects of polysaccharides on intestinal mucosal function and gut microbiota are gradually becoming a focus of research for many researchers.

## Mechanisms of bile acids in biological activity in humans

As one of the important steroid molecules, bile acids (BAs) are synthesized by cholesterol metabolism in the liver. In the human body, cholesterol is often converted into cholic acid (CA) and chenodeoxycholic acid (CDCA) through two pathways—classical and alternative pathways. Subsequently, CA and CDCA are acylated with glycine or taurine in the liver to form conjugated bile salts, which are stored in the gallbladder and then released from the bile duct into the duodenum. Cholesterol 7α-hydroxylase (cytochrome P450 family 7 subfamily A member 1, CYP7A1) and mitochondrial sterol 27 hydroxylase (cytochrome P450 family 27 subfamily A member 1, CYP27A1) are key enzymes involved in the two pathways of bile acid synthesis (30). After being discharged into the intestine with bile, a portion of primary bile acids is reabsorbed by intestinal epithelial cells and returned to the liver through the portal vein, while another portion is metabolized into secondary bile acids (including lithocholic acid and deoxycholic acid) by intestinal microbiota. In the intestine, the conjugated BAs undergo uncoupling, oxidation, isomerization, and dehydroxylation reactions to convert primary BAs into secondary BAs (31). During the process of hepatic intestinal circulation, approximately 95% of BAs are reabsorbed and transported from the intestine to the liver through specific transporters at the end of the ileum. Then, the free BAs are recombined with taurine or glycine and released into the intestine through bile ducts.

Bile acids are produced through cholesterol catabolism and play an important role in maintaining whole-body cholesterol homeostasis (32). Cholic acid (CA) accounts for approximately 31% of total human bile acids. Evidence from modern pharmacological studies demonstrates that CA acts as a signaling molecule to bind to bile acid receptors, regulate bile acid metabolism, and restore bile acid homeostasis (33). CA activates the PPARα -UGT pathway and compensates for the loss of bile acid transport and synthesis function in SHP knockout mice, ultimately helping to maintain overall intestinal structure and bile acid homeostasis (34).

At the same time, CA can also act as a regulator of glucose, lipids, and energy metabolism to improve metabolic-related inflammation (35). Bile acids regulate a wide range of complex symbiotic metabolic networks by activating various bile acid receptors, including glucose, lipids, steroids, and metabolism, as well as regulating energy homeostasis, thus profoundly affecting the host’s metabolism and immune function (36). The fibroblast growth factor 21 (FGF21) pathway is considered an important pathway for regulating glucose and lipid metabolism. Acetyl CoA carboxylase-1 (ACC1) and hormone-sensitive lipase (HSL) are key enzymes that control the supply of long-chain fatty acid precursors for liver triglyceride (TG) synthesis. On the one hand, CA can reduce the activity of HSL and ACC1 through the FGF21 pathway and ultimately play a role in regulating glucose and lipid metabolism. On the other hand, CA reduces the expression of MIP-1 α, IL-10, and IL-8, upregulates the expression of PPARα, and downregulates the expression of LXRα to exert their anti-inflammatory effects (37–39).

In addition, CA exerts its novel activities by reducing oxidative stress in brain cells and playing a neuroprotective role in brain injury due to its good blood–brain barrier permeability and antioxidant properties (14). Defects or mutations in key enzyme genes of bile acid metabolism often cause disorders of bile acid anabolism. Then, they affect the body’s bile acid metabolism homeostasis and lead to the occurrence of some diseases, such as Zellweger spectrum disorders and cerebrotendinous xanthomatosis (40). CA can interact with the peripheral anion sites of acetylcholinesterase to inhibit its activity and reduce the deposition of amyloid plaques, thus playing a therapeutic role in AD (41). In addition, CA plays a neuroprotective role by promoting the release of brain-derived neurotrophic factor (BDNF), which is beneficial for activating the BDNF Trkb—PI3K/Akt and BDNF Trkb MAPK/ERK signaling pathways, reducing cell damage caused by oxidative stress, and inhibiting cell apoptosis (42).

## Metabolism of bile acids by gut microbiota

Gut microbiota is considered one of the important organs of the human body. It is widely involved in the metabolism of bile acids. It is manifested in the role of modifying the chemical structure of bile acid, reabsorption, and detoxification. The mechanisms of gut microbiota in the metabolism of bile acids mainly include early dissociation, dehydroxylation, dehydrogenation, and desulfurization. Firstly, primary bile acids derived from the host are early dissociated by bile salt hydrolases (BSHs), which are mainly found in lactic acid bacteria, *Clostridium*, *Bifidobacterium*, *Enterococcus*, *Listeria*, and *Clostridium* (43). Secondly, *Clostridium* is involved in the 7α-dehydroxylation of bile acids, which converts primary bile acids into secondary bile acids (44). 3) Three kinds of hydroxysteroid dehydrogenases (HSDHs)—3α-HSDH, 7α-HSDH, and 12α-HSDH—expressed by gut microbiota are involved in bile acid hydroxyl oxidation and specific epimerization to convert hydrophobic toxic bile acids into non-toxic water-soluble ursodeoxycholic acid (45). 3α-HSDH is expressed by *Clostridium perfringens*, *Peptostreptococcus, Pseudomonas*, and others. 7α -HSDH is widely found in *Bacteroides*, *Clostridium*, *Escherichia coli,* and *Ruminococcus*. 12α-HSDH is mainly found in *Clostridium* (46). 4) Some gut microbiota, such as *Clostridium S2,* produce sulfatase to increase the desulfurization of bile acids and promote the reabsorption of bile acids (46).

## The roles of polysaccharides in the regulation of the intestines

### Regulatory effects of polysaccharides on gut microbiota

Gut microbiota is considered one of the important components that participates in regulating certain biological activities in humans through different pathways (47). Polysaccharides often serve as important energy sources for gut microbiota and are thus involved in regulating its composition and function. Gut microbiota often encodes a variety of carbohydrate-activated enzymes to catalyze the degradation of polysaccharides into short-chain fatty acids, such as acetic acid, propionic acid, and butyric acid, thereby providing necessary energy for gut microbiota (48). Research demonstrates that polysaccharides from *Arctium lappa* (300 mg/kg, gavage for 25 days) significantly increase the abundance of *Lactobacillius, Alistipes, Odoribacter, and Phascolarctobacterium* and reduce the abundance of *Bacteroides*. This induces a significant increase in the content of short-chain fatty acids in the intestine and alleviates systemic inflammation caused by intraperitoneal injection of lipopolysaccharide in mice (49). The chemical structure of the polysaccharides used in this experiment is reported to be a type of inulin-neoseries fructan (neokestose), which consists of (2→1)-β-D-Fruf linked to a terminal (2→1)-α-d-glucopyranose at the non-reducing end, with (2→6)-β-D-Fruf branching on its backbone (50). Yang et al. conducted a study on the effect of flaxseed polysaccharides (50–150 g/kg, gavage for 8 weeks) in mice with metabolic syndrome induced by a high-fat diet. They found that the proportion of *Ackermann* and *Bifidobacterium* increased with the decreased abundance of *Oscillospira* and *Odoribacteraceae*. This is considered to be beneficial for reducing fat accumulation and the occurrence and development of metabolic syndrome (51, 52). The chemical composition of the polysaccharides in this experiment is not reported.

### The effect of polysaccharides on intestinal barrier function

Tight junctions between cells play an important role in maintaining the integrity of the intestinal barrier and function by regulating paracellular transport. Intercellular tight junction-related proteins mainly include ZO family proteins, occludin, claudins, immunoglobulin superfamily proteins, and others (53, 54). A previous study investigated the effect of *Dendrobium officinale* polysaccharides (DOPSs; 50–200 mg/kg, gavage for 6 weeks) on colitis in rats induced by DSS. The study found that DOPSs enhance the expression of ZO-1 and occludin in the intestine, therefore effectively recovering intestinal barrier function and reducing inflammation caused by colitis in the rats (55). The monosaccharide composition of the DOPSs used in the experiment was composed of mannose, glucose, and arabinose, with a molar ratio of 5.55:1:0.12 (average molecular weight 393.8 kDa) (56). In another study, it was demonstrated that longan pulp acidic polysaccharides (LPIa, LPIIa, and LPIIIa; 100–400 mg/kg, gavage for 4 weeks) could increase the expression of ZO-1, claudin-1, occludin, and the adhesion junction protein E-cadherin to protect the intestinal mucosa from being injured by cyclophosphamide (57). Among all the reported polysaccharides, it is noted that LPIa has specific structure characteristics, including a porous surface structure, a high molecular weight (1.47 × 10^5^ Da), and two specific glycosidic linkages of α-Araf-(1→ and →5)-α-Araf-(1→. It is deduced that the special structural features of LPIa significantly contribute to the protection of the intestinal barrier (57).

### Regulatory effect of polysaccharides on intestinal digestive enzyme activity

Polysaccharides also play a key role in regulating the activity of intestinal digestive enzymes, optimizing the intestinal environment, and improving intestinal diseases, which influence the digestion and absorption of food in humans. It was reported that constipation in mice induced by diphenoxylate was significantly relieved by the administration of polysaccharides from *Spirulina platensis* (PSP; 50–200 mg/kg, gavage for 1 week) through the restoration of intestinal xylanase and protease activities to normal levels (58). The chemical composition of PSP in this article is not presented. In another study, it was reported that PSP is composed of →2)-α-L-Rhap-(1→, →4)-β-D-Manp-(1→, →6)-β-D-Glcp-(1→, →4)-β-Xylp-(1→, →3)-β-L-Araf-(1→, and →2)-β-L-Fucp-(1→, (59). In addition, a study demonstrated that polysaccharides from *Dendrobium officinale* (560 mg/kg, gavage for 1 week) are effective in restoring intestinal xylanase, amylase, and protease activities and in decreasing intestinal cellulase activity in mice with spleen deficiency constipation (60). The core structure of polysaccharides from *Dendrobium officinale* is composed mainly of glucose and mannose (Manp:Glcp = 2.01:1.00–8.82:1.00), along with galactose, xylose, arabinose, and rhamnose in varying molar ratios (61). Taken together, these results indicate that the regulation of intestinal digestive enzyme activity is also closely related to the regulation of gut microbiota.

## The interactions between polysaccharides and bile acids

Polysaccharides often exert their regulatory effects on human physiological functions and for the treatment of diseases by regulating intestinal function, including gut microbiota, intestinal physiological barriers, and the intestinal digestive enzymes system. In addition, polysaccharides are involved in regulating the metabolism of bile acids, thereby indirectly contributing to the regulation of human physiological functions and the treatment of some diseases. The regulation of bile acids by polysaccharides is mainly through three pathways. Firstly, polysaccharides directly bind some bile acids, which leads to a decrease in reabsorption and an increase in the excretion of bile acids. Secondly, polysaccharides directly regulate the expression of intestinal transporters, thereby affecting the reabsorption and excretion of bile acids in the intestines. Lastly, the composition and structure of gut microbiota involved in the activities of synthesis, reabsorption, metabolism, and excretion of bile acids are regulated by polysaccharides. The interactions between polysaccharides and bile acids in regulating human physiological functions and treating diseases are summarized as follows.

### Polysaccharides directly bind to fats, cholesterol, and bile acids

Polysaccharides often play an important role in the regulation of lipid metabolism and energy consumption by directly binding to fats, cholesterol, and bile acids in the intestine. Hence, it is considered that some polysaccharides are effective in the regulation of diseases related to metabolism, such as diabetes and obesity. In an *in vitro* intestinal simulation experiment, research showed that pectic polysaccharides from loquat (*Eriobotrya japonica*) leaves exhibit a higher affinity to fat/cholesterol and bile acids compared to carboxymethyl cellulose and cholestyramine, which may be developed as food for the prevention and treatment of obesity and type 2 diabetes (52). The major monosaccharide components of the polysaccharides from loquat leaves are rhamnose, galacturonic acid, arabinose, and galactose. In addition, the novel binding capacity of the polysaccharides from loquat leaves to fat/cholesterol and bile acids is correlated with their molecular weights (4.307 ~ 5.101 × 10^6^ Da), molecular weight distributions, and degree of esterification (52). In the same experiment, it was also found that polysaccharides from okra (*Abelmoschus esculentus*) exhibit an obvious binding capacity to fat/cholesterol and bile acids, which may be useful for increasing the excretion of fat, cholesterol, and bile acids (62). In addition, polysaccharides often directly bind bile acids to inhibit the reabsorption and excretion of bile acids. The bile acid-binding capacities of polysaccharides from *Caulerpa lentillifera* were investigated, and it was found that the binding ability of the sub-fraction WCLP-70 to cholic acid, deoxycholic acid, glycocholic acid, and taurocholic acid is 68.1, 36.1, 74.9, and 72.3%, respectively (63). Researchers have also found that crude polysaccharides from *Laminaria japonica* (LP-A8) present a strong binding ability to cholic acid, glycocholic acid, and taurocholic acid, with binding values of 68.29, 81.99, and 161.72%, respectively. It is deduced that the highly-branched structure, abundant sulfate, fucose, and galactose in the chemical composition, and the denser interconnected macromolecule network in the molecular morphology of the polysaccharides (LP-A8) contribute significantly to its bile acid-binding capacity (64). Polysaccharides from *Momordica charantia* L., which are mainly composed of arabinose, galactose, glucose, xylose, and mannose, also exhibit a strong binding capacity to cholic acid and chenodeoxycholic acid *in vitro* (65). In addition, research has demonstrated that the stigma maydis polysaccharide (SMP-1) exhibits a strong binding capacity to both taurocholic acid sodium (65.96–83.64%) and glycodeoxycholic acid sodium (71.76–103.50%) *in vitro*, while also lowering total cholesterol, triglyceride, and low-density lipoprotein cholesterol levels in hyperlipidemic mice (66). Chemical structure analysis has shown that the SMP-1 is composed of D-mannose, L-rhamnose, D-glucose, D-galactose, L-arabinose, D-xylose, and D-galacturonic acid, with a molar ratio of 1.00:0.21:1.41:1.44:0.70:0.44:0.56. These components are bonded by (1→6) and (1→3) linkages, with various monosaccharides distributed in the main and side chains (66). Taken together, these results suggest that although various polysaccharides have been tested for their bile acid-binding capacities *in vitro* in previous studies, it should be noted that their actual binding abilities to bile acids *in vivo* have been scarcely investigated. The real mechanisms underlying the anti-hyperlipidemic effects through direct binding to bile acids *in vivo* should be further explored.

### Polysaccharides regulate bile acids by modulating intestinal transporters

Apart from directly binding to bile acids, polysaccharides also participate in regulating the expression of some intestinal transporters to facilitate the reabsorption and excretion of bile acids. It has been found that the expression of the FXR and bile acid transporters ASBT and MRP2 in the ileum, as well as the FXR, OSTα/β, and MRP3 in the cecum, are significantly increased by the daily consumption of apple pectin for piglets (67). This leads to an increased amount of excreted bile acids in feces and a decreased serum cholesterol level. In another *in vivo* experiment, it was noted that cholesterol accumulation was significantly decreased, along with improved cholesterol metabolism, by daily administration of Haw pectin penta-oligogalacturonide (300 mg/kg BW, 4 weeks) to mice fed with a high-cholesterol diet. A mechanism study reported that Haw pectin penta-oligogalacturonide treatment significantly reduces the expression of the FXR in the intestine of mice and suppresses intestinal bile acid reabsorption (68). It has also been found cellulose from sweet potato residues (100 mg/kg BW, 28 days) is beneficial for lipid metabolism in rats, inhibiting body weight gain, food intake, plasma lipids, and hepatic lipids. Mechanisms investigation has demonstrated that the ileal apical sodium-dependent bile acid transporter and intestinal bile acid-binding protein are increased by treatment with this substance in hypolipidemic rats (69).

### Polysaccharides regulate the metabolism of bile acids by modulating gut microbiota

In fact, gut microbiota is widely involved in the metabolism of numerous nutrients in humans and plays an important role in the entire process of life. In addition to affecting the expression of receptors related to intestinal bile acid metabolism, orally administrated polysaccharides also widely interfere with gut microbiota to regulate bile acid metabolism. It was reported that polysaccharides from *Grifola frondosa* (900 mg/kg, 8 weeks) significantly increased the hepatic expression of the bile acid synthase CYP7A1 and the bile salt export pump (BSEP) in diabetic mice, which is beneficial for the enhancement of bile acids (BAs) synthesis and excretion in the liver. A correlation analysis indicated that *Roseburia*, *Lachnoclostridium*, *Lachnospiraceae*_*NK4AB6_group*, *Rikenella*, *Bacteroides,* and *Alistipes* positively correlate to the mRNA levels of *CYP7A1* and *BSEP*, while *Streptococcus*, *Staphylococcus*, *Enterococcus,* and *Aerococcus* negatively correlate with BSEP mRNA levels (70). Huang et al. found that sulfated polysaccharides from *Gracilaria lemaneiformis* (GLP, 60 mg/kg, 6 weeks) significantly accelerated the conversion of cholesterol to bile acids by promoting the expression of *LxRα* and *CYP7A1* genes in high-fat fed mice. It was found that the serum concentration of chenodeoxycholic acid and deoxycholic acid is negatively correlated with the relative abundances of *Alistipes*, *Prevotellaceae*_*UCG-001,* and *Corprococcus*, while ursodeoxycholic acid and tauroursodeoxycholic acid are positively correlated with the relative abundances of *Lachnospiraceae_NK4A136_group* and *Roseburia*. The chemical composition of the polysaccharides in the experiment was reported to be composed of galactose, glucose, fucose, and mannose, with a molar ratio of 9.16: 6.57: 1.00: 0.61. The glycosidic linkages of GLP are as follows: 84→3,4)-Fucp-(1→, →3,4,6)-Galp-(1→, →4)-Glcp-(1→, →4,6)-Manp-(1→, →6)-Glcp-(1→,85→6)-Galp-(1→ and Galp-(1→ with the ratio of 0.36: 1.36: 0.26: 0.13: 36.58: 54.36: 6.96 (71).

**Figure 1:**
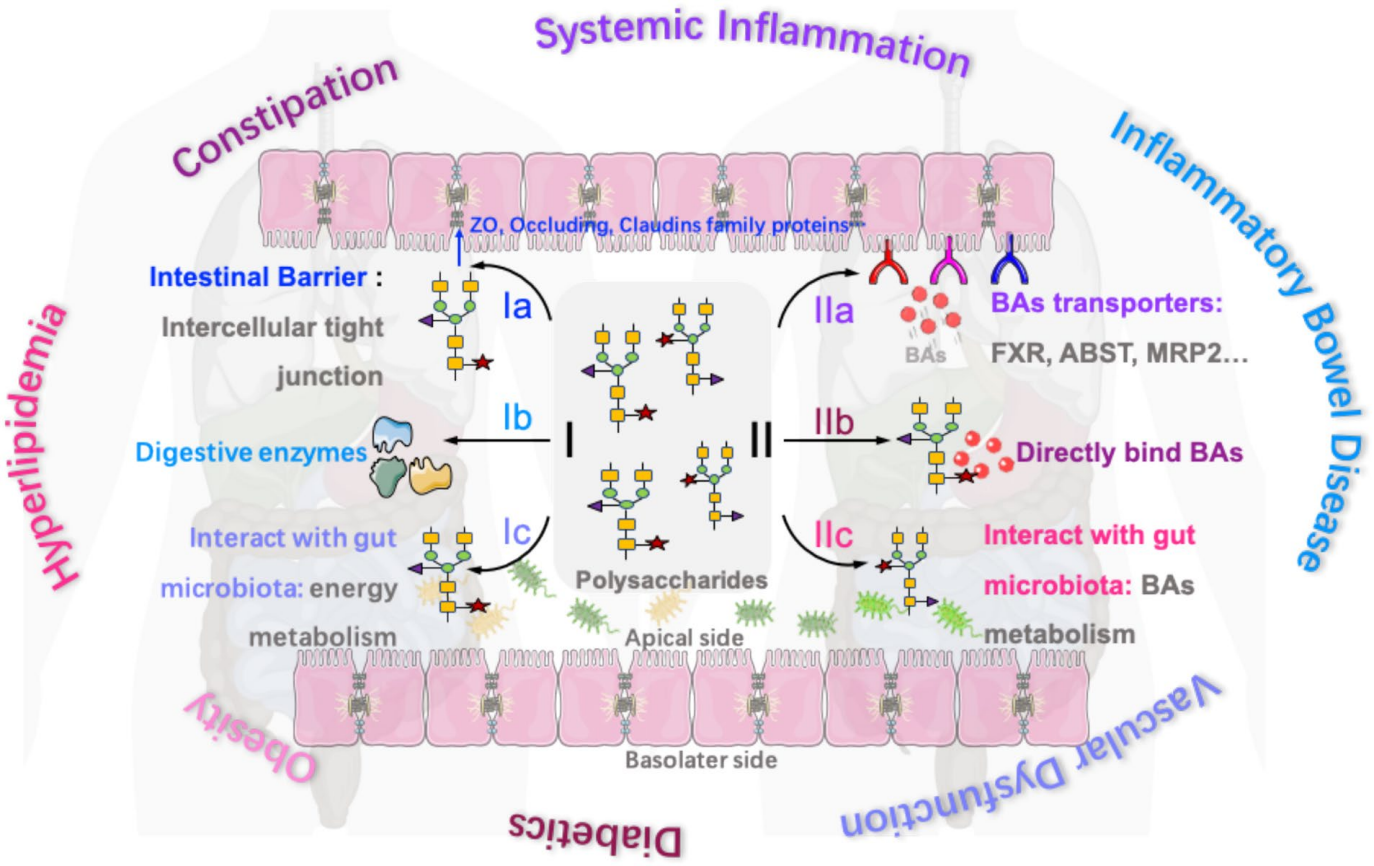
Mechanisms of bioactive polysaccharides in the regulation of intestinal function and bile acids for the treatment of various diseases (Ia, polysaccharides regulate the expression of intercellular tight junction proteins in the intestines for treating inflammatory bowel disease and constipation; Ib, polysaccharides regulate the production of some digestive enzymes in the intestines for treating constipation and aiding nutrient digestion; Ic, polysaccharides interact with gut microbiota for regulating energy metabolism and treating hyperlipidemia and obesity; IIa, polysaccharides regulate the expression of some bile acids transporters (such as FXR, ABST, and MRP2) to regulate bile acids resorption in the intestine for treating systemic inflammation, obesity, and diabetes; IIb, some polysaccharides also directly bind to certain bile acids to inhibit bile acids resorption for treating hyperlipidemia and obesity; and IIc, polysaccharides interact with gut microbiota for regulating bile acid metabolism and treating diabetics and obesity).

Polysaccharides from green microalga *Chlorella pyrenoidosa* (150–300 mg/kg, 8 weeks) were also reported to accelerate the metabolism of cecal total bile acids and short-chain fatty acids in hyperlipidemia rats. The results indicated that total bile acids in the rat cecum are positively correlated with *Ruminococcus*_1, *Coprococcus*_1, *Peptococcus,* and *Acetatifactor*. The chemical structure of polysaccharides from green microalga *Chlorella pyrenoidosa* is identified as follows: the main glycosidic bonds include 1,2-linked-α-L-Fucp, 1,4-linked-α-L-Rhap, 1,4-linked-β-L-Araf, 1-linked-α-D-Glcp, 1,3-linked-β-D-GlcpA, 1,4-linked-β-D-Xylp, and 1,3,6-linked-β-D-Manp, with a molecular weight of 5.63 × 10^6^ Da (72). Catry et al. reported that inulin-type fructan treatment (250 mg/mouse/day, 2 weeks) successfully replenished the abundance of *Akkermansia* and decreased the abundance of bacterial taxa involved in secondary BA synthesis in mice with vascular dysfunction. It was found that the content of cholic acid and chenodeoxycholic acid (primary bile acid) in the blood and cecum of mice were increased, while the content of lithocholic acid and deoxycholic acid (secondary bile acid) was decreased, which facilitated the improvement of endothelial dysfunction. Meanwhile, lithocholic acid and deoxycholic acid were positively correlated with the abundances of *Ruminococcaceae* and Lachnospiraceae (73). A summary of the mechanisms of bioactive polysaccharides in the regulation of intestinal function and bile acids for the treatment of various diseases is presented in Figure 1.

From previous studies, we have found that polysaccharides effectively regulate gut microbiota and bile acid metabolism, thus playing a therapeutic role in some diseases. However, further research should focus more on the crosstalk between orally administrated polysaccharides, gut microbiota, and bile acid metabolism. Whether polysaccharides directly regulate the composition or enzyme activity of gut microbiota, thereby indirectly affecting the metabolism of bile acids; whether polysaccharides affect the composition or content of intestinal bile acids, thereby indirectly regulating the composition and biological activity of gut microbiota; or whether polysaccharides participate in more complex regulatory processes— these questions still need further investigation. Further and more in-depth research on bioactive polysaccharides should be conducted to investigate the exact chemical composition of polysaccharides and their corresponding activities in the regulation of intestine function and bile acid metabolism. Answering these kinds of questions will be more conducive to the development of bioactive polysaccharides for human health.

## Conclusion

Polysaccharides regulate bile acid synthesis, reabsorption, metabolism, and excretion through the intestine, which can provide new insights for the development and mechanistic research of polysaccharide-based drugs for the treatment of diseases caused by abnormal bile acid metabolism, such as hyperlipidemia, diabetes, cardiovascular diseases, and non-alcoholic fatty liver diseases, as well as for formulating new targeted treatment strategies. The structure of polysaccharides is complex due to the differences in monosaccharide composition, glycosidic bonds, and degree of polymerization. The structure of flora is easily altered by external or internal factors, and a variety of substances can be obtained after the polysaccharide is degraded by carbohydrate-active enzymes. The dynamic changes of the gut-bile acid-host axis are complex. People’s understanding of the degradation mechanism of polysaccharides, the end products of bacterial metabolism, the regulation of microbiota structure, and the impact of metabolites on the body is still limited. The mechanisms by which polysaccharides regulate bile acids require further research to be fully understood.
